# Acute myeloid leukemia and myelodysplastic neoplasms: clinical implications of myelodysplasia-related genes mutations and *TP53* aberrations

**DOI:** 10.1007/s44313-024-00044-4

**Published:** 2024-12-18

**Authors:** Hyunwoo Kim, Ja Young Lee, Shinae Yu, Eunkyoung Yoo, Hye Ran Kim, Sang Min Lee, Won Sik Lee

**Affiliations:** 1 Department of Laboratory Medicine, Inje University Busan Paik Hospital, Inje University College of Medicine, Busan, Korea; 2 Paik Institute for Clinical Research, Inje University Busan Paik Hospital, Busan, Korea; 3 Department of Laboratory Medicine, Inje University Haeundae Paik Hospital, Inje University College of Medicine, Busan, Korea; 4 Department of Internal Medicine, Inje University Busan Paik Hospital, Inje University College of Medicine, Busan, Korea

**Keywords:** Acute myeloid leukemia, Gene mutations, International Consensus Classification, World Health Organization

## Abstract

**Purpose:**

The fifth World Health Organization (WHO) classification (2022 WHO) and International Consensus Classification (ICC) of myeloid neoplasms have recently been published. In this study, patients were reclassified according to the revised classification and their prognoses were analyzed to confirm the clinical utility of the new classifications.

**Methods:**

We included 101 adult patients, 77 with acute myeloid leukemia (AML) and 24 with myelodysplastic neoplasms (MDS), who underwent bone marrow aspiration and next-generation sequencing (NGS) between August 2019 and July 2023. We reclassified the patients according to the revised criteria, examined the differences, and analyzed the prognosis using survival analysis.

**Results:**

According to the 2022 WHO and ICC, 23 (29.9%) patients and 32 (41.6%) patients were reclassified into different groups, respectively, due to the addition of myelodysplasia-related (MR) gene mutations to the diagnostic criteria or the addition of new entities associated with *TP53* mutations. The median overall survival (OS) of patients with AML and MR gene mutations was shorter than that of patients in other AML groups; however, the difference was not significant. Patients with AML and *TP53* mutation had a significantly shorter OS than the other AML group (*p* = 0.0014, median OS 2.3 vs 10.3 months). They also had significantly shorter OS than the AML and MR mutation group (*p* = 0.002, median OS 2.3 vs 9.6 months).

**Conclusion:**

The revised classifications allow for a more detailed categorization based on genetic abnormalities, which may be helpful in predicting prognosis. AML with *TP53* mutations is a new ICC category that has shown a high prognostic significance in a small number of cases.

**Supplementary Information:**

The online version contains supplementary material available at 10.1007/s44313-024-00044-4.

## Introduction

The World Health Organization (WHO) classification of myeloid neoplasms has been revised several times to improve our understanding of the molecular features of this disease [1]. The development of molecular genetic technology has advanced our understanding of myeloid neoplasms by adding distinct groups to their classification, such as acute myeloid leukemia (AML) with genetic abnormalities [2–4]. The commercialization of next-generation sequencing (NGS) has introduced genetic information into the diagnostic criteria for AML and myelodysplastic neoplasms (MDS). Recent studies have provided a detailed census of genes mutated in myeloid neoplasms; thus, the number of gene mutations incorporated into AML diagnosis and risk stratification has increased [5, 6]. The fifth edition of the WHO classification (2022 WHO) and the International Consensus Classification (ICC) of myeloid neoplasms have been published [7, 8]. Changes included lowering the blast threshold that defines AML and renaming “myelodysplastic syndrome” as “myelodysplastic neoplasm.”

One of the largest differences between the revised fourth WHO classification (2016 WHO) and the 2022 WHO/ICC classification is the change in the diagnostic criteria for AML associated with myelodysplasia. In the 2016 WHO guidelines, the main diagnostic criteria for AML with myelodysplasia-related changes (AML-MRC) are morphological changes in the bone marrow (BM), history of MDS, and chromosomal abnormalities [9]. In the 2022 WHO, morphological dysplasia alone is excluded from the criteria, and mutations in eight myelodysplasia-related (MR) genes (*ASXL1, BCOR, SF3B1, EZH2, SRSF2, STAG2, U2AF1*, and *ZSZR2*) are included [8]. *RUNX1* mutations were added to the ICC criteria in addition to the eight MR genes [7].

Another significant difference is the addition of MDS and AML groups associated with *TP53* mutations [7, 8]. In the 2022 WHO report, subtype MDS with biallelic *TP53* inactivation (MDS-bi*TP53*) was identified when there were two or more *TP53* mutations or when there was one mutation with evidence of *TP53* copy number loss [8]. A subtype of MDS with mutated *TP53* (MDS-*TP53*) was added to ICC. AML with mutated *TP53* (AML-*TP53*) was added only to ICC [7]. Subsequently, the 2022 European Leukemia Net (ELN) risk stratification system was revised to include new diagnostic classifications [4]. Besides *ASXL1* and *RUNX1*, which were already classified as adverse in the 2017 ELN risk stratification, six other MR gene mutations were newly classified as adverse.

These changes have advanced our understanding of the molecular genetic characteristics of AML and MDS and have helped us apply this knowledge to clinical diagnosis and therapeutic strategies. Several studies have primarily focused on reclassification, examining the differences in diagnostic criteria between the 2016 WHO classification and the two new classifications [9–11]. However, recent studies have targeted the prognostic effects of specific AML subtypes such as AML, myelodysplasia-related (AML-MR), and AML-*TP53* [12–14]. A study in Korea found that patients with AML-MR, according to the 2022 WHO guidelines, had a shorter overall survival (OS), similar to that of patients with AML-MRC, according to the 2016 WHO guidelines [15]. A study of a small group of patients with MDS reported that those with *TP53* mutations had a worse prognosis than those without *TP53* mutations [16].

In this study, we reclassified patients with myeloid neoplasms who were initially classified according to the 2016 WHO at our institution according to the revised criteria. We identified gene mutation rates in AML and MDS and performed survival analyses for the subgroups, particularly focusing on AML with MR genes and *TP53* mutations. The clinical outcomes of these groups were compared with those of the other AML subgroups to evaluate the clinical usefulness of the new classifications.

## Materials and methods

### Patient selection and data collection

This study included all patients aged ≥ 18 years who had a BM examination and targeted NGS between August 2019 and July 2023. During this period, 288 patients underwent BM examination with suspicion of AML or MDS, of whom 105 were tested using NGS at the time of the initial diagnosis. Electronic medical records were retrospectively reviewed with respect to each patient’s demographic data and laboratory findings, including BM examination results, treatment information, and survival outcomes. After excluding four patients who subsequently relapsed and received the same NGS results as before, 101 patients were enrolled. This study was approved by the Institutional Review Board of Inje University, Busan Paik Hospital (2023–10-026), which waived the requirement for informed consent. After reviewing the data, patients were reclassified according to the 2022 WHO and ICC guidelines [7, 8].

### Molecular tests including targeted next-generation sequencing

For fusion gene detection, RNA was isolated from BM using a QIAamp RNA Blood Mini Kit (Qiagen, Hilden, Germany). Multiplex reverse transcription polymerase chain reaction was performed using the HemaVision-28N Panel (DNA Diagnostics, Risskov, Denmark).

NGS was performed using the MiSeq Dx sequencing platform (Illumina, San Diego, CA, USA). Forty-eight genes that were associated with AML and MDS were identified (Supplementary Table S1). The targeted specimen was genomic DNA isolated from BM aspirates, and the target enrichment method was hybridized with oligonucleotide probes. The panel version and bioinformatics pipeline were NGB-Wet-V2.0 and NGB-DNA-somatic-V1.3, respectively. The sequence was aligned to the human reference genome GRCh37. The average depth of coverage was 592.8X. Among the NGS test results, gene mutations with Tier 3, unknown clinical significance, were excluded [17].

### Karyotyping

BM samples were processed after 24 and 48 h of unstimulated culture using GTL-banding (Giemsa banding using trypsin and Leishman staining). The band resolution was 300 to 400 bands. The karyotypes were interpreted according to the International System for Human Cytogenetic Nomenclature [18].

### Statistical analysis

Chi-square and Fisher’s exact tests were used to detect differences in the gene mutation distribution in each classification system. Patients with AML were divided into prognostic groups based on ELN risk stratification [4]. The OS was defined as the period from the time of diagnosis to death or the end of follow-up. The endpoint was October 31, 2023. The OS of the patients in each group was analyzed using the Kaplan–Meier curve and log-rank test. Multivariate analysis of OS was performed using the Cox proportional hazards model. All statistical analyses were performed using MedCalc (version 12.4; MedCalc Software, Ostend, Belgium) and R ( version 4.4.1; RStudio version 2024. 04.2–764) at *p* < 0.05. significant.

### Human Ethics and Consent to Participate declarations

This study was a retrospective medical record analysis using general clinical characteristics, molecular genetic test results, and patient charts. No unnecessary blood collections or tests were performed. Personal identification information was anonymized and managed. This study was conducted in accordance with the principles of the Declaration of Helsinki and approved by the Institutional Review Board of Inje University, Busan Paik Hospital, Busan, Korea (2023–10-026).

## Results

### Reclassification of AML and MDS based on the revised criteria

Seventy-seven patients with AML and 24 patients with MDS were enrolled in this study. The median age of the AML group was 67 years (range, 19–88), and that of the MDS group was 73 years (range, 54–86). Fifty-eight patients were male (57.4%).

**Fig. 1:**
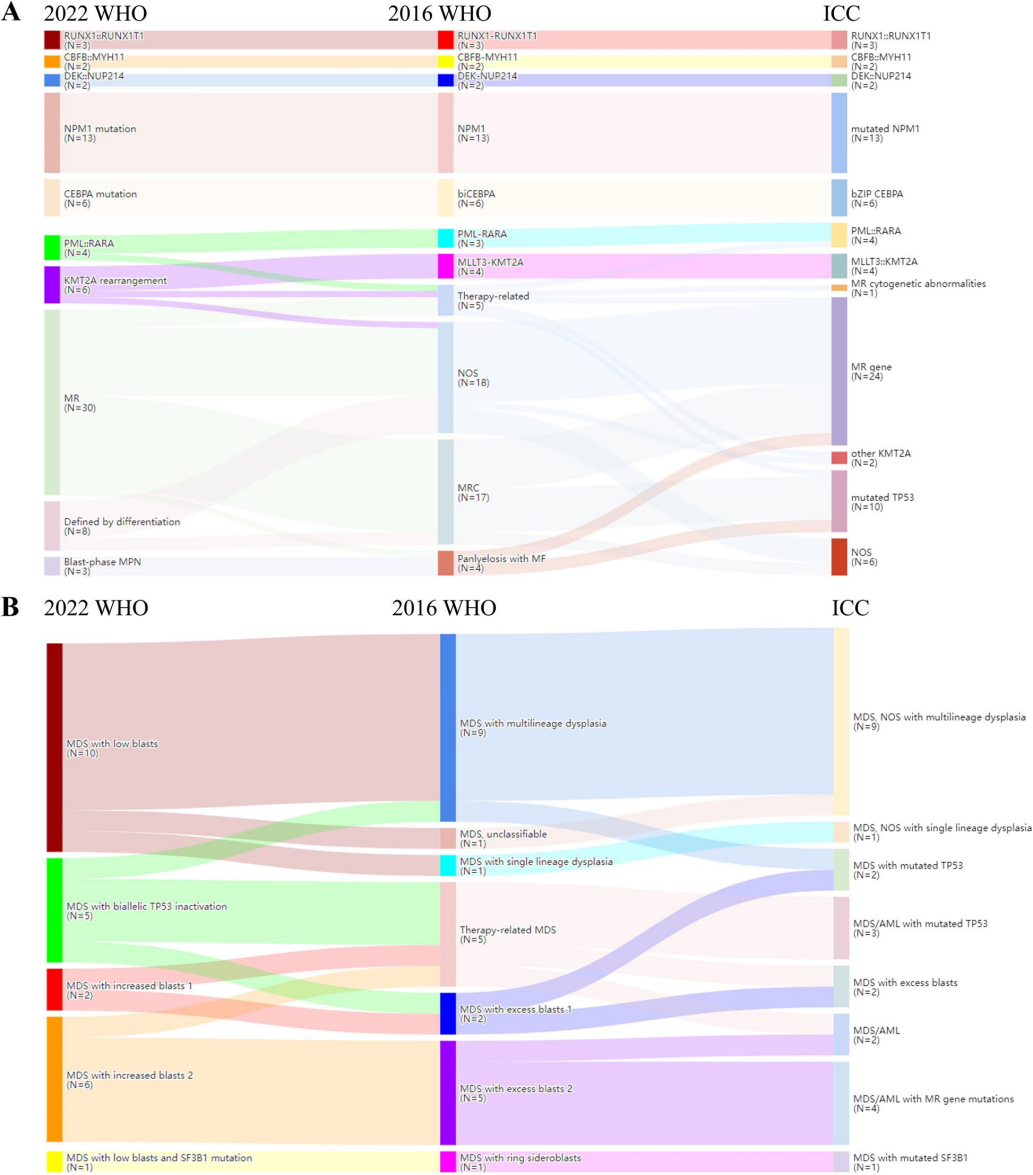
Sankey diagram showing the reclassification of patients with (**A**) acute myeloid leukemia and (**B**) myelodysplastic neoplasm, from the 2016 World Health Organization (WHO) to the 2022 WHO and International Consensus Classification

Among the 77 patients with AML, 23 (29.9%) and 32 (41.6%) patients were reclassified into other groups based on the 2022 WHO and ICC, respectively (Fig. 1A). Using the revised criteria, 33 patients (42.9%) with AML and recurrent genetic abnormalities were classified the same as the 2016 WHO, with only the group names changed (Table 1). Of the 17 patients (22.1%) with AML-MRC in the 2016 WHO, 15 were reclassified as AML-MR based on the 2022 WHO. Seven of these patients harbored *TP53* mutations and were reclassified as AML-*TP53,* according to the ICC. Of the 18 patients (23.4%) diagnosed with AML not otherwise specified (NOS) according to the 2016 WHO, 11 (61.1%) were reclassified as AML-MR based on the 2022 WHO. For the ICC, 13 (72.2%) were reclassified as AML-MR because of the differences in MR gene mutations between the two classifications. One patient with t(1;11)(p32;q23) was reclassified as having AML with *KMT2A* rearrangement (AML-*KMT2A*) using the 2022 WHO criteria and AML with other *KMT2A* rearrangements using the ICC.

**Table 1:** Reclassification of acute myeloid leukemia and myelodysplastic neoplasm based on the 2022 WHO and ICC

	**2016 WHO**	**N**	**%**	**2022 WHO**	**N**	**%**	**ICC**	**N**	**%**
**AML**	*RUNX1-RUNX1T1*	3	3.9%	*RUNX1::RUNX1T1*	3	3.9%	*RUNX1::RUNX1T1*	3	3.9%
	*CBFB-MYH11*	2	2.6%	*CBFB::MYH11*	2	2.6%	*CBFB::MYH11*	2	2.6%
	*PML-RARA*	3	3.9%	*PML::RARA*	4	5.2%	*PML::RARA*	4	5.2%
	*DEK-NUP214*	2	2.6%	*DEK::NUP214*	2	2.6%	*DEK::NUP214*	2	2.6%
	*MLLT3-KMT2A*	4	5.2%	*KMT2A* rearrangement	6	7.8%	*MLLT3::KMT2A*	4	5.2%
							Other *KMT2A*	2	2.6%
	*NPM1*	13	16.9%	*NPM1* mutation	13	16.9%	mutated *NPM1*	13	16.9%
	bi*CEBPA*	6	7.8%	*CEBPA* mutation	6	7.8%	bZIP *CEBPA*	6	7.8%
	Myelodysplasia-related changes	17	22.1%	Myelodysplasia-related	30	39.0%	MR gene	24	31.2%
							MR cytogenetic abnormalities	1	1.3%
	Panmyelosis with myelofibrosis	4	5.2%	Blast-phase MPN	3	3.9%	NA		
	NA			NA			mutated *TP53*	10	13.0%
	Not otherwise specified	18	23.4%	Defined by differentiation	8	10.4%	Not otherwise specified	6	7.8%
	Therapy-related	5	6.5%	NA			NA		
**MDS**	Single lineage dysplasia	1	4.2%	Low blasts	10	41.7%	NOS with single lineage dysplasia	1	4.2%
	Multilineage dysplasia	9	37.5%				NOS with multilineage dysplasia	9	37.5%
	Excess blasts-1	2	8.3%	Increased blasts-1	2	8.3%	Excess blasts	2	8.3%
	Excess blasts-2	5	20.8%	Increased blasts-2	6	25.0%	MDS/AML	2	8.3%
							MDS/AML with MR gene mutations	4	16.7%
							MDS/AML with *TP53*	3	12.5%
	Ring sideroblasts	1	4.2%	Low blasts and *SF3B1*	1	4.2%	*SF3B1*	1	4.2%
	NA			bi*TP53*	5	20.8%	mutated with *TP53*	2	8.3%
	Unclassifiable	1	4.2%	NA			NA		
	Therapy-related	5	20.8%	NA			NA		

*Abbreviations: WHO* World Health Organization, *ICC* International Consensus Classification, *AML* acute myeloid leukemia, *bZIP* basic leucine zipper, *MR* myelodysplasia-related, *NA* not applicable, *MDS* myelodysplastic neoplasm

In the 2022 WHO and ICC guidelines, therapy-related AML (t-AML) was removed and added as an additional qualifier to other AML categories. Of the five patients who were diagnosed with t-AML in the 2016 WHO, three were reclassified as AML-MR, one as acute promyelocytic leukemia (APL), and one as AML-*KMT2A* according to the 2022 WHO. In the ICC, two patients diagnosed with t-AML according to 2016WHO were reclassified as the same as the 2022 WHO, whereas three patients with AML-MR according to the 2022 WHO were reclassified as AML-*TP53*, AML-MR and AML with MR cytogenetic abnormalities in the ICC, respectively. Patients with panmyelosis and fibrosis were also excluded. Three patients had a history of myeloproliferative neoplasm (MPN). Therefore, they were reclassified as blast-phase MPN by the 2022 WHO. In ICC, these patients were reclassified as AML-MR and AML-*TP53* based on the presence of genetic mutations.

Among the 24 patients with MDS, 7 (29.2%) and 12 (50.0%) patients were reclassified into other groups based on the 2022 WHO and ICC, respectively (Fig. 1B). Despite the adjustment of the BLAST threshold in the 2022 WHO and ICC schemes, no MDS cases were reclassified as AML because of the absence of defined genetic abnormalities or rare gene fusion. In ICC, one patient with MDS with excess blasts 2 (MDS-EB2) and one patient with t-MDS, according to the 2016 WHO criteria, were reclassified into the MDS/AML group, and four patients with MDS-EB2 were reclassified into MDS/AML with MR gene mutations. One patient with MDS with multilineage dysplasia (MDS-MD) and one with MDS with excess blast 1 (MDS-EB1) in the 2016 WHO classification were reclassified as having MDS-bi*TP53* based on the 2022 WHO. Based on ICC, they were reclassified as MDS-*TP53*. Three patients with t-MDS were reclassified as having MDS/AML with mutated *TP53* (MDS/AML-*TP53*) in the ICC and MDS-bi*TP53* in the 2022 WHO. One patient with MDS, unclassifiable (MDS-U), was reclassified as having MDS with low blasts (MDS-LB) by the 2022 WHO, and MDS, NOS with multilineage dysplasia in the ICC. MDS with ring sideroblasts (MDS-RS) was revised, and the name was changed to MDS with low blasts and *SF3B1* mutations (MDS, LB, and *SF3B1*) by the 2022 WHO and MDS with mutated *SF3B1* (MDS-*SF3B1*) in the ICC.

### Genetic mutation landscape in AML and MDS

**Fig. 2:**
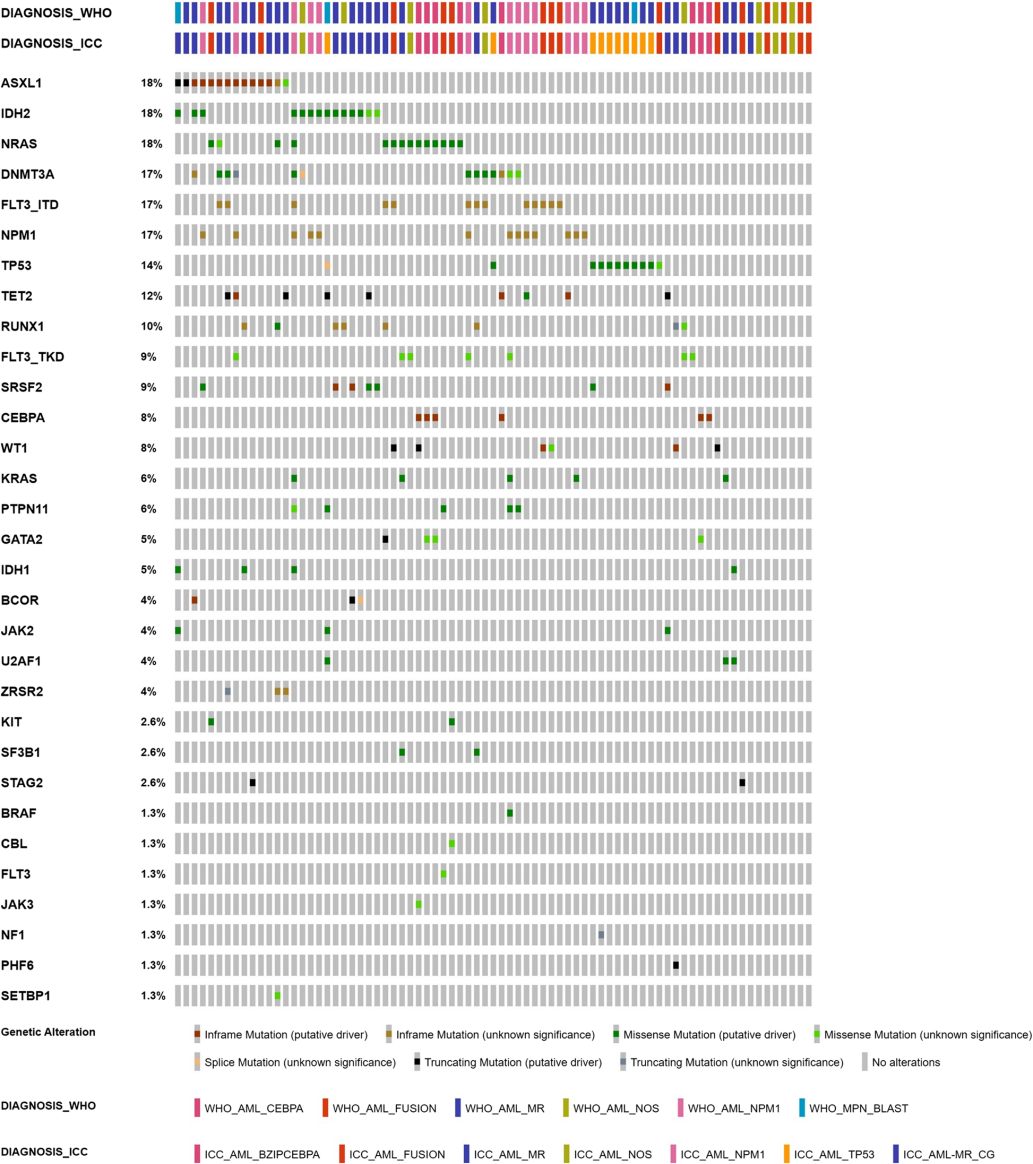
Molecular landscape of patients with acute myeloid leukemia

Approximately 84.2% (85/101) of all patients had mutations in 29 genes. In total, 89.6% (69/77) of the patients with AML and 66.7% (16/24) of those with MDS had 211 mutations (median 2, range 0 – 9 per patient) and 36 mutations (median 2, range 0—7 per patient), respectively (Fig. 2). Among 69 patients with AML and mutations, 16 (20.8%) had 2 mutations and 36 patients (52.2%) had ≥ 3 mutations. The most common AML mutations were *ASXL1*, *IDH2* and *NRAS* (14 patients, 18.2%), followed by *DNMT3A*, *FLT3*-ITD, and *NPM1* (13 patients, 16.9%). *ASXL1* and *IDH2* mutations were predominantly identified in patients with AML-MR, whereas *NRAS* mutations were frequently detected in patients with AML, defining genetic abnormalities in the 2022 WHO. *DNMT3A* and *FLT3*-ITD mutations were most commonly observed in patients with AML harboring *NPM1* mutations. In a survival analysis of gene mutations present in more than 10% of patients with AML, a statistically significant difference was detected in median OS based on only *FLT3-ITD* and *TP53* mutations (*p* < 0.05; median OS 7.2 and 2.3 months with mutation vs. 11.7 and 10.3 months without mutation, respectively).

In patients with MDS, the most frequently observed gene mutations were *ASXL1* and *TP53*, each found in five patients (20.8%; Supplementary Fig. 1). Mutations in *ASXL1*, *RUNX1*, and *STAG2* were significantly more common in patients with MDS and increased blasts (MDS-IB) than in those with MDS-LB in the 2022 WHO (*p* < 0.05; Table 2).

**Table 2:** Patient characteristics and gene mutations in myelodysplastic neoplasm

	All MDS	2022 WHO			
		Low blasts	Increased blasts	bi*TP53*	*p*
Patients	24	11	8	5	
Sex, male: female	15:9	6:5	6:2	3:2	*0.656*
Age, years (range)	73(54–86)	73 (56–83)	70 (54–85)	70 (58–80)	*0.930*
Laboratory findings, median value					
White blood cell, × 10^9^/L, median (range)	2.48 (0.31–34.12)	2.44 (0.31–3.78)	2.27 (1.05–34.12)	3.46 (1.70–7.87)	*0.406*
Hemoglobin, g/dL, median (range)	8.3 (4.1–12.7)	9.1 (5.5–11.0)	6.9 (5.9–12.7)	7.5 (4.1–9.7)	*0.537*
Platelet, × 10^9^/L, median (range)	67.5 (6.0–264.0)	77.0 (6.0–259.0)	57.5 (10.0–264.0)	62.0 (21.0–102.0)	*0.931*
Blasts in peripheral blood, %, median (range)	0 (0–9)	0 (0–1)	1 (0–9)	3 (0–7)	*0.004*
Blasts in bone marrow, %, median (range)	4.8 (0–19.2)	2.2 (0–4.5)	14.5 (5.1–19.2)	13.0 (2.6–13.4)	< *0.001*
Cytogenetics					
Abnormal karyotype, N (%)	11 (45.8%)	4 (36.4%)	3 (37.5%)	4 (80.0%)	*0.226*
Complex karyotype, N (%)	6 (25.0%)	2 (18.2%)	0 (0%)	4 (80.0%)	*0.003*
Gene mutations					
N, median (range)	2 (0–7)	0 (0–3)	4 (0–7)	0 (0–2)	*0.040*
Tumor suppressor					
* TP53*	5 (20.8%)	0	0	5 (100%)	< *0.001*
Transcription factors (except MR gene)					
* RUNX1*	4 (16.7%)	0	4 (42.9%)	0	*0.008*
* CEBPA*	2 (8.3)	0	2 (28.6%)	0	*0.113*
Myelodysplasia related genes					
* ASXL1*	5 (20.8%)	0	5 (62.5%)	0	*0.002*
* BCOR*	3 (12.5%)	1 (9.1%)	2 (25.0%)	0	*0.373*
* SF3B1*	2 (8.3%)	1 (9.1%)	0	0	*0.725*
* SRSF2*	2 (8.3%)	0	2 (25.0%)	0	*0.113*
* STAG2*	4 (16.7%)	0	4 (50.0%)	0	*0.008*
* U2AF1*	1 (4.2%)	0	1 (12.5%)	0	*0.352*
* ZRSR2*	1 (4.2%)	1 (9.1%)	0	0	*0.540*
DNA methylation					
* DNMT3A*	3 (12.5%)	2 (18.2%)	1 (12.5%)	0	*0.595*
* IDH2*	1 (4.2%)	0	1 (12.5%)	0	*0.352*
* TET2*	1 (4.2%)	0	1 (12.5%)	0	*0.352*
RNA helicase					
* DDX41*	2 (8.3%)	2 (18.2%)	0	0	*0.276*

*Abbreviations: MDS* myelodysplastic neoplasm, *WHO* World Health Organization, *MR* myelodysplasia

### Patient characteristics and clinical outcomes in AML with MR gene mutations based on 2022 WHO classification

Among the 25 patients with AML and MR gene mutations, three had gene fusions, two had a previous history of MPN, and one had an *NPM1* mutation. Six patients were not classified as having AML-MR according to the 2022 WHO. Thirty patients were classified as AML-MR according to the 2022 WHO, and MR gene mutations were present in 66.7% (20/30). The remaining nine patients had complex karyotypes, and one patient had a history of MDS.

In our study, *ASXL1* mutations (*N* = 9, 30.0%) were the most frequent among MR genes, followed by *SRSF2* mutations (*N* = 6, 20.0%). Although *RUNX1* mutation is included in MR genes only in the ICC, most of the *RUNX1* mutations (75%, 6/8) were found in patients with AML-MR in the 2022 WHO. Patients with AML-MR were significantly older, had lower white blood cell (WBC) counts, and were more likely to have complex karyotypes than those in the other groups (Table 3). When analyzing the frequency of gene mutations between the AML-MR and other groups, *TP53* and *SRSF2* mutations showed a significantly higher rate in patients with AML-MR (*p* < 0.05).

**Table 3:** Patient characteristics and gene mutations in acute myeloid leukemia

	All AML	2022 WHO			ICC			
		AML-MR	AML-others	*p*	AML-TP53	AML-MR	AML-others	*p*
Patients	77	30	47		10	25	42	
Sex, male: female	43:34	20:10	23:24	*0.196*	8:2	14:11	21:21	*0.229*
Age, years (range)	67 (19–88)	71 (32–87)	62 (19–88)	*0.002*	69.5 (57–87)	71 (32–83)	62 (19–88)	*0.034*
Laboratory findings, median value								
WBC, × 10^9^/L, median (range)	5.94 (0.81–231.1)	3.25 (1.03–224.18)	13.71(0.81–231.05)	*0.005*	3.29 (1.57–26.92)	3.33 (1.02–224.18)	15.27 (0.81–231.05)	*0.009*
Hemoglobin, g/dL, median (range)	8.0 (2.6–15.0)	7.9 (2.7–11.0)	8.3 (2.6–15.0)	*0.154*	8.3 (5.3–9.8)	7.8 (2.7–11.0)	8.3 (2.6–15.0)	*0.647*
Platelet, × 10^9^/L, median (range)	40.0 (3.0–344.0)	39.5 (3–143)	42.0 (6–344)	*0.711*	36.0 (3.0–48.0)	51.0 (4.0–143.0)	35.0 (6.0–344.0)	*0.458*
Blasts in PB, %, median (range)	27 (0–96)	18.5 (0–90)	30 (0–96)	*0.339*	4.5 (0–90)	27.0 (0–88)	30.0 (0–96)	*0.218*
Blasts in BM, %, median (range)	57.4 (13.7–92.7)	52.0 (21.2–92.7)	60.2 (13.7–91.8)	*0.137*	43.7 (23.2–90.3)	52.0 (21.2–92.7)	60.2 (13.7–91.8)	*0.511*
Cytogenetics								
Abnormal karyotype, N (%)	45 (58.4%)	20 (66.7%)	25 (53.2%)	*0.458*	8 (80.0%)	13 (52.0%)	24 (57.1%)	*0.060*
Complex karyotype, N (%)	14 (18.2%)	13 (43.3%)	1 (2.1%)	< *0.001*	8 (80.0%)	5 (20.0%)	1 (2.4%)	< *0.001*
Gene mutations								
N, median (range)	2 (0–9)	2.5 (0–6)	2 (0–9)	*0.557*	1 (1–6)	3 (0–6)	2 (0–9)	*0.082*
RAS pathway-related								
* NRAS*	14 (18.2%)	4 (13.3)	10 (21.3)	*0.563*	0	4 (16.0%)	10 (23.8%)	*0.202*
* KRAS*	5 (6.5%)	2 (6.7)	3 (6.4)	*0.671*	0	2 (8.0%)	3 (7.1%)	*0.667*
* KIT*	2 (2.6%)	0	2 (4.3)	*0.682*	0	0	2 (4.8%)	*0.425*
* FLT3*-ITD	13 (16.9%)	4 (13.3)	9 (19.1)	*0.725*	0	4 (16.0%)	9 (21.4%)	*0.264*
* FLT3*-TKD	7 (9.1%)	1 (3.3)	6 (12.8)	*0.319*	0	2 (8.0%)	5 (11.9%)	*0.487*
* PTPN11*	5 (6.5%)	0	5 (10.6)	*0.170*	1 (10.0)	0	4 (9.5%)	*0.276*
Tumor suppressor								
* TP53*	11 (14.3%)	8 (26.7)	3 (6.4)	*0.032*	10 (100.0)	0	1 (2.3)	< *0.001*
* PHF6*	1 (1.3%)	1 (3.3)	0	*0.820*	0	1 (4.0%)	0	*0.349*
* WT1*	6 (7.8%)	1 (3.3)	5 (10.6)	*0.465*	0	1 (4.0%)	5 (11.9%)	*0.311*
Transcription factors (except MR gene)								
* RUNX1*	8 (10.4%)	6 (20.0)	2 (4.3)	*0.068*	0	8 (32.0%)	0	< *0.001*
* CEBPA*	6 (7.8%)	0	6 (12.8)	*0.109*	0	0	6 (14.3%)	*0.067*
* SETBP1*	1 (1.3%)	1 (3.3)	0	*0.820*	0	1 (4.0%)	0	*0.349*
* GATA2*	4 (5.2%)	1 (3.3)	3 (6.4)	*0.951*	0	1 (4.0%)	3 (7.1%)	*0.624*
Myelodysplasia related								
* ASXL1*	14 (18.2)	9 (30.0)	5 (10.6)	*0.065*	0	10 (43.5)	4 (9.5%)	*0.002*
* BCOR*	3 (3.9)	3 (10.0)	0	*0.108*	0	3 (12.0%)	0	*0.039*
* SF3B1*	2 (2.6)	2 (6.7)	0	*0.290*	0	2 (8.0%)	0	*0.118*
* SRSF2*	7 (9.1)	6 (20.0)	1 (2.1)	*0.024*	1 (10.0)	5 (20.0%)	1 (2.4%)	*0.051*
* STAG2*	2 (2.6)	1 (3.3)	1 (2.1)	*0.682*	0	1 (4.0%)	1 (2.4%)	*0.791*
* U2AF1*	3 (3.9)	2 (6.7)	1 (2.1)	*0.689*	1 (10.0)	2 (8.0%)	0	*0.148*
* ZRSR2*	3 (3.9)	3 (10.0)	0	*0.108*	0	3 (12.0%)	0	*0.039*
DNA methylation								
* DNMT3A*	13 (16.9)	5 (16.7)	8 (17.0)	*0.786*	1 (10.0)	4 (16.0%)	8 (19.0%)	*0.782*
* IDH1*	4 (5.2)	2 (6.7)	2 (4.3)	*0.951*	0	3 (12.0%)	1 (2.4%)	*0.168*
* IDH2*	14 (18.2)	6 (20.0)	8 (17.0)	*0.978*	1 (10.0)	8 (32.0%)	5 (11.9%)	*0.092*
* TET2*	9 (11.7)	4 (13.3)	5 (10.6)	*0.996*	1 (10.0)	4 (16.0%)	4 (9.5%)	*0.716*
Risk group by ELN 2022 guideline								
Favorable, N (%)	20 (26.0)	0	20 (42.6%)	< *0.001*	0	0	20 (47.6%)	< *0.001*
Intermediate, N (%)	18 (23.4)	0	18 (38.3%)		0	0	18 (42.9%)	
Adverse, N (%)	39 (50.6)	30 (100%)	9 (19.1%)		10 (100%)	25 (100%)	4 (9.5%)	

*Abbreviations: AML* acute myeloid leukemia, *WHO* World Health Organization, *ICC* International Consensus Classification, *MR* myelodysplasia-related, *PB* peripheral blood, *BM* bone marrow, *ELN* European Leukemia Net

**Fig. 3:**
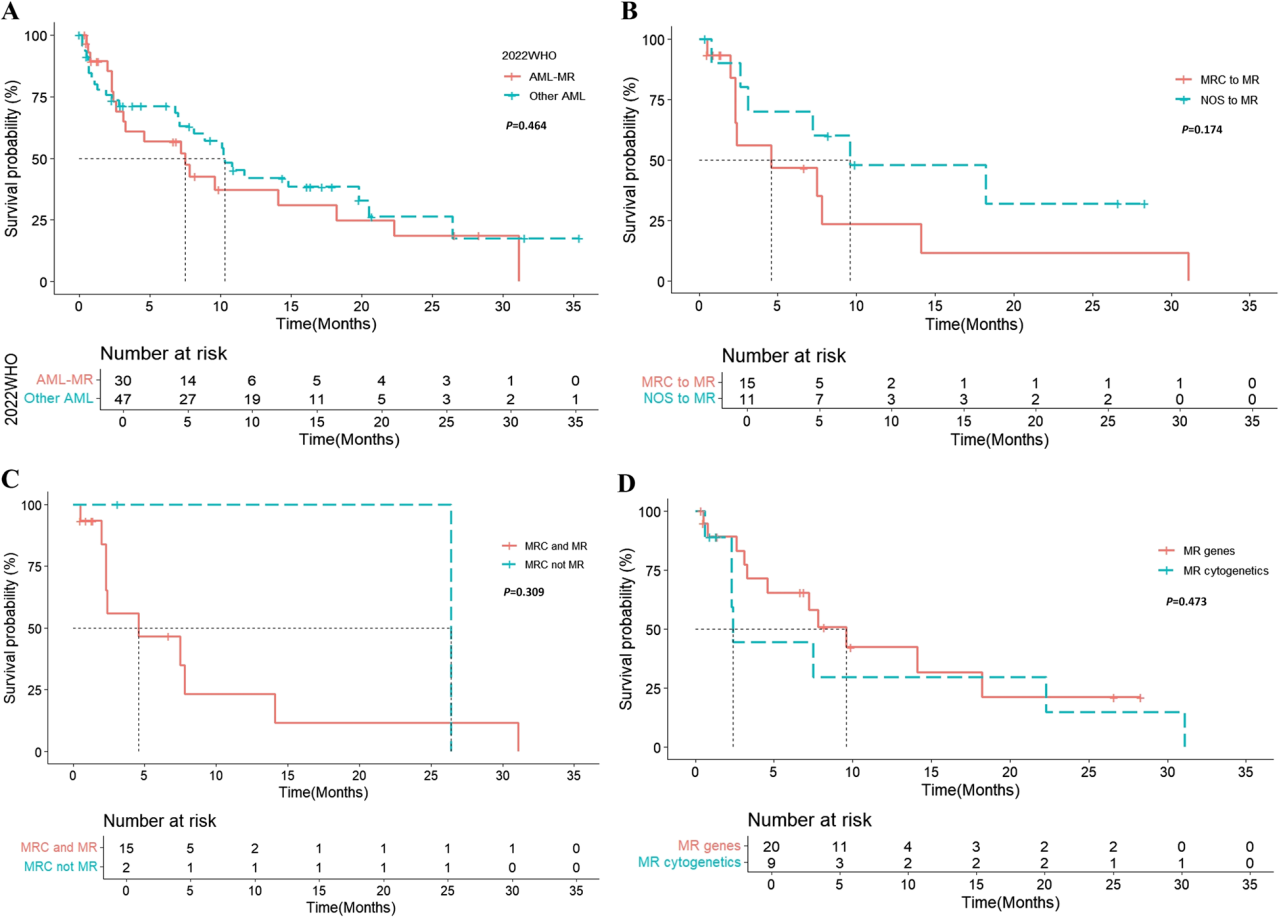
Survival analysis of patients with acute myeloid leukemia (AML) and myelodysplasia-related (MR) gene mutations based on the 2022 WHO classification. (**A**) Survival outcomes between patients with AML and MR mutations and those with other types of AML. (**B**) Survival outcomes between groups of patients with AML with myelodysplastic changes (MRC), classified according to the 2016 WHO and MR gene mutation criteria in the 2022 WHO, and those with AML, not otherwise specified, according to the 2016 WHO and AML with MR gene mutation in the 2022 WHO. (**C**) Survival outcomes between groups of patients with AML with MRC based on the 2016 WHO and AML with MR gene mutations in the 2022 WHO, and those with AML with MRC in the 2016 WHO but without MR gene mutation in the 2022 WHO. (D) Survival outcomes between groups of patients of AML with MR gene mutations and cytogenetic abnormalities

Based on the 2022 ELN risk stratifications, all patients with AML-MR were classified into the adverse group. The median OS of the AML-MR group was shorter than that of the other AML groups (*p* = 0.464; median OS 7.5 vs. 10.3 months, Fig. 3A). The median OS of patients with AML-MRC in the 2016 WHO and AML-MR group in the 2022 WHO was shorter than that of patients with AML-NOS in the 2016 WHO classified as AML-MR in the 2022 WHO (*p* = 0.174; median OS 4.6 vs 9.6 months, Fig. 3B). Particularly, the AML-MRC group in the 2016 WHO and the AML-MR group in the 2022 WHO demonstrated shorter OS than the group classified as AML-MRC in the 2016 WHO, but not as AML-MR in the 2022 WHO (Fig. 3C). When examining the OS of patients with AML, those with cytogenetic abnormalities showed a relatively shorter median OS than those with mutations in MR (*p* = 0.473; median OS 2.4 vs. 9.6 months, Fig. 3D). The median OS of patients with *SRSF2* mutation (3.85 months) was the shortest among the patients with MR gene mutations; however, statistical significance was not reached because of the small sample size (Table 4).

**Table 4:** Comparison of survival outcomes in patients with acute myeloid leukemia based on the presence of myelodysplasia-related gene mutations

Gene	N	Median OS (months)	Range
*ASXL1*	14	7.8	0.5–28.3
*BCOR*	3	NA	8.2–26.6
*SF3B1*	2	10.4	2.6–18.2
*SRSF2*	7	3.85	0.4–8.2
*STAG2*	2	6.7	3.3–10.1
*U2AF1*	3	NA	0.6–6.7
*ZRSR2*	3	7.8	7.2–14.1

*Abbreviations*: *OS* overall survival, *NA* not applicable

### Patient characteristics and clinical outcomes in patients with AML and MDS with TP53 mutations based on the ICC classification

**Fig. 4:**
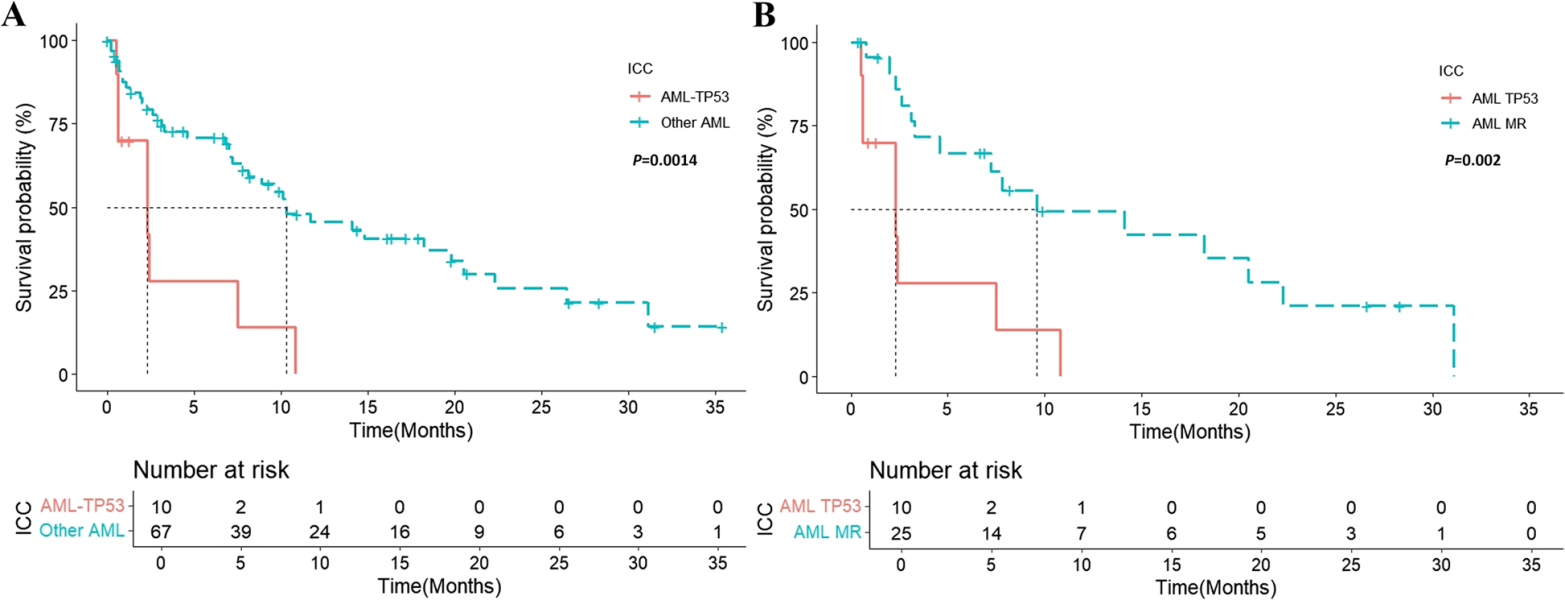
Survival analysis of patients with AML and *TP53* mutations based on the international consensus classification. **A** Survival outcomes between patients in AML with *TP53* mutations and other AML group. **B** Survival outcomes between patients in AML with *TP53* mutation and AML with myelodysplasia-related gene mutations

*TP53* mutations were found in 11 patients with AML (14.3%) and 5 with MDS (20.8%). The median variant allelic frequencies of *TP53* mutations in AML and MDS were 53.97% and 38.16%, respectively. Biallelic *TP53* mutations were observed in only four patients with MDS. Ten patients with AML and *TP53* mutations were classified as having AML-*TP53,* according to the ICC. One patient with *MLLT3-KMT2A* fusion was classified as having AML with *MLLT3::KMT2A*. In the ICC, AML is divided into three groups: AML-*TP53*, AML-MR, and AML. The AML-*TP53* and AML-MR groups were significantly older, had lower WBC counts, and exhibited higher rates of complex karyotypes than the other groups. A significant negative correlation was identified between *TP53* mutations and the expression of other genes. According to the ICC, 25 patients with AML-MR showed a significantly higher frequency of mutations in *ASXL1, BCOR*, and *ZRSR2* (*p* < 0.05). Ten patients with AML-*TP53* were classified into the adverse group according to the 2022 ELN risk stratification. The median OS of the AML-*TP53* group was significantly shorter than that of the non-AML-*TP53* group (*p* = 0.0014, median OS 2.3 vs. 10.3 months, Fig. 4A). They also had a significantly shorter OS than the AML-MR group (*p* = 0.002; median OS 2.3 vs. 9.6 months, Fig. 4B). In multivariate analysis, *TP53* mutations were the only factor significantly associated with the overall survival of AML (hazard ratio 9.372, *p* = 0.007) when age, WBC count, and the presence of complex karyotypes and MR genes were adjusted (Supplementary Table S2). Patients with MDS and *TP53* mutations had shorter median OS than patients without *TP53* mutations (*p* = 0.1792; median OS 9.5 vs. 21.9 months).

## Discussion

We conducted a retrospective study of the newly revised classification of myeloid neoplasms, focusing on two key genetic alterations, MR genes and *TP53* mutations, at a single institution to identify differences and assess their clinical utility.

The major changes in the 2022 WHO guidelines were the exclusion of morphological dysplasia based on the diagnostic criteria for AML-MR and the addition of eight MR gene mutations [8]. In this study, 11 patients were reclassified from AML and NOS to AML-MR by the 2022 WHO guidelines, resulting in a larger AML-MR group than the AML group. Based on the ICC, the *RUNX1* mutation was added to the AML-MR criteria [7], and 13 patients in the AML and NOS groups were reclassified as having AML-MR. Differences in chromosomal abnormalities were observed in the diagnostic criteria for myelodysplasia-related AML included in the 2022 WHO and ICC, such as 11q deletion, monosomy 13, or 13q deletion in the 2022 WHO criteria and trisomy 8 or 20q deletion in the ICC criteria. In this study, one patient was reclassified as having AML with MR cytogenetic abnormalities based on an abnormal karyotype.

Twenty-three (29.9%) and 32 (41.6%) patients were reclassified based on the 2022 WHO and ICC criteria, respectively. In contrast, a single-center study in Korea by Lee et al. [16] reported higher reclassification rates: 31.9% for 2022 WHO and 57.4% for ICC. This discrepancy is likely because their study excluded patients with AML with balanced translocations according to the 2016 WHO guidelines. Direct comparisons across institutions are challenging because reclassification rates can vary depending on the type of neoplasm, such as AML or MDS, and whether the target gene or fusion transcript is included in the analysis. Notably, the group with the most significant changes was the AML, NOS group in the 2016 WHO, while AML, MR was the largest subgroup in the 2022 WHO and ICC, a trend also observed in other studies [14, 16, 19, 20].

In a similar single-center study, Zhou et al. [14] reported that the most common mutations in AML-MR were *TP53*, *RUNX1* and *ASXL1*. Our results are consistent with these findings; *ASXL1* and *TP53* mutations were most frequently found in patients with AML-MR. Among the MR genes, mutations in *SRSF2* were the most common after *ASXL1*, similar to the frequency reported in a Korean study by Park et al. [15]. In survival analysis, patients with AML-MR showed worse outcomes. In a comparative analysis of survival rates between patients classified as AML-MRC in the 2016 WHO but not as AML-MR in the 2022 WHO guideline and those classified as both AML-MRC and AML-MR in the 2022 WHO guideline, revealed that MR-associated cytogenetic abnormalities and the presence of MR genes were more significant prognostic factors than morphological abnormalities [21, 22]. Additionally, patients with AML-MR with only cytogenetic abnormalities had shorter survival outcomes than those with only MR gene mutations. This finding aligns with those of previous reports, although statistical significance was not observed because of the small sample sizes. Previous studies have reported significantly shorter OS in patients with AML and *ASXL1*, *SRSF2*, and *ZRSR2* mutations among MR genes than in those without these mutations [15, 22, 23]. In this study, the *ZRSR2* mutation was detected in only three patients with AML-MR and was found together with the *ASXL1* mutation in all cases. Consequently, despite its low detection frequency among MR genes, it significantly influences survival outcomes when co-occurring with *ASXL1*. In our study, although none of the MR gene mutations showed a significant difference in the median OS, there were differences in the median OS based on the MR genes. Although *RUNX1* mutations are only included as MR genes in ICC, a shorter survival time was observed in patients with AML and *RUNX1* mutations than in patients with other MR gene mutations in this study, and it was the second most frequent mutation after *TP53* in AML-MR in the 2022 WHO. Large-scale studies are needed to assess the differences in the frequency and prognosis of MR gene mutations, including *those in RUNX1*.

Another major change in the 2022 WHO and ICC classifications is the new category of *TP53* mutations. *TP53* mutations are commonly associated with complex karyotype, negative correlation with other gene mutations, and poor prognosis in AML and MDS [7, 24, 25]. In our study, patients with AML and *TP53* mutations exhibited similar characteristics and poor survival outcomes. In the 2022 WHO, a distinct category of myeloid neoplasms with mutated *TP53* was defined for MDS but not for AML. Therefore, the AML-MR group included patients with *TP53* mutations that frequently occur in patients with complex chromosomal abnormalities. MR gene mutations are also associated with poor survival in AML; however, survival analysis between the AML-MR and other AML groups did not show a statistically significant difference. In contrast, ICC separated the AML-MR and AML-*TP53*, revealing a significant difference in survival outcomes between the two groups. This suggests that the AML-*TP53* classification may provide a more precise stratification of patients with poor prognosis based on ICC. MR genes have been reported to have better prognostic significance than *TP53* mutations, and the results of our study are consistent with this [12].

In this study, patients with AML-*TP53* presented with extremely poor survival and a median OS of only 2.3 months. This was significantly lower than the survival rates reported in previous studies on AML and *TP53* mutations [13]. For patients with high-risk AML, intensive chemotherapy, followed by allogeneic hematopoietic stem cell transplantation (allo-HSCT), is recommended as a potentially curative approach [26]. However, patients with AML harboring *TP53* mutations exhibit a lower probability of achieving remission, and allo-HSCT has not been shown to improve OS [27]. In this study, five patients with AML-*TP53* received intensive chemotherapy consisting of daunorubicin or idarubicin combined with cytarabine. Of these, only two achieved complete remission, and only one underwent all-HSCT. The remaining five patients were treated with hypomethylating agents, and one patient died before a final diagnosis was made. Although the median OS of patients who underwent HSCT was longer than that of patients who received chemotherapy alone (10.8 vs 2.3 months), the difference was not statistically significant. Therefore, an optimal treatment strategy for patients with AML-*TP53* is yet to be established.

This study has several limitations. First, although treatment guidelines may affect prognosis, patients were not classified based on their treatment regimens, and treatment modifications were not accounted for according to age. Consequently, it was not possible to perform a survival analysis stratified by treatment modality. Additionally, because the data were derived from a single institution with a short observation period, some subgroup analyses were limited by the small number of cases that showed statistical significance. Finally, we used only NGS data, without fluorescence in situ hybridization, to identify *TP53* mutations and related chromosomal abnormalities.

In conclusion, the two newly revised classification criteria allowed us to further categorize patients diagnosed with AML and NOS into more specific subgroups, such as AML-MR and AML-*TP53*. While AML-MR is already known to be associated with poor survival, this study reinforces the idea that AML-MR with cytogenetic abnormalities has an even worse prognosis than those with only MR-associated gene mutations. Additionally, the newly introduced AML-*TP53* group in ICC showed highly significant prognostic value, even in a small number of patients. As more data are accumulated and analyzed, these revised classification criteria will become more useful for predicting prognosis and facilitating a more detailed categorization of myeloid neoplasms at the gene mutation level.

## Supplementary Information

Supplementary Material 1: Supplementary Fig.1. Molecular landscape of patients with myelodysplastic neoplasm.

Supplementary Material 2.

## Data Availability

Data is provided within the manuscript or supplementary information files.
