# Correction: Acute myeloid leukemia and myelodysplastic neoplasms: clinical implications of myelodysplasia‑related genes mutations and TP53 aberrations

**DOI:** 10.1007/s44313-025-00054-w

**Published:** 2025-01-13

**Authors:** Hyunwoo Kim, Ja Young Lee, Shinae Yu, Eunkyoung Yoo, Hye Ran Kim, Sang Min Lee, Won Sik Lee

**Affiliations:** 1 Department of Laboratory Medicine, Inje University Busan Paik Hospital, Inje University College of Medicine, Busan, Korea; 2 Paik Institute for Clinical Research, Inje University Busan Paik Hospital, Busan, Korea; 3 Department of Laboratory Medicine, Inje University Haeundae Paik Hospital, Inje University College of Medicine, Busan, Korea; 4 Department of Internal Medicine, Inje University Busan Paik Hospital, Inje University College of Medicine, Busan, Korea

**Correction: Blood Res 59, 41 (2024)**

**https://doi.org/10.1007/s44313-024-00044-4**

Following publication of this article, it was noticed that the author name Shinae Yu appeared incorrectly as Sinae Yu.

The author group has been updated above and the original article has been corrected.

