# Supplementary figures and images for: Acute myeloid leukemia and myelodysplastic neoplasms: clinical implications of myelodysplasia-related genes mutations and TP53 aberrations
Source: Blood Res. 2024 Dec 18;59(1):41. doi: 10.1007/s44313-024-00044-4 (PMC11655781; doi:10.1007/s44313-024-00044-4)

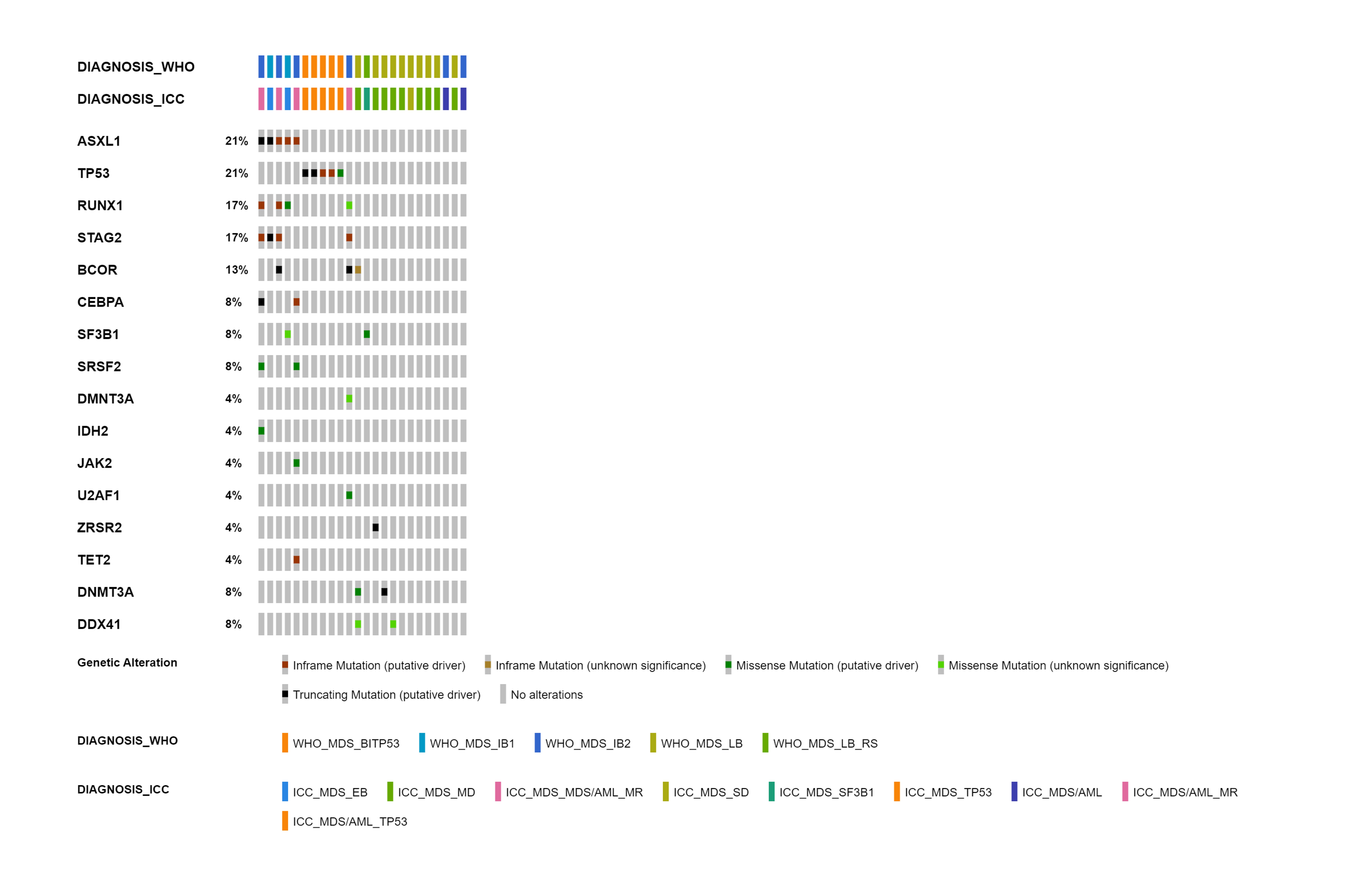

Supplement: Supplementary file 1 — Supplementary Material 1: Supplementary Fig.1. Molecular landscape of patients with myelodysplastic neoplasm. [file 44313_2024_44_MOESM1_ESM.jpg]
