# Supplementary material for: Acute myeloid leukemia and myelodysplastic neoplasms: clinical implications of myelodysplasia-related genes mutations and TP53 aberrations
Source: Blood Res. 2024 Dec 18;59(1):41. doi: 10.1007/s44313-024-00044-4 (PMC11655781; doi:10.1007/s44313-024-00044-4)
Supplement: Supplementary file 2 — Supplementary Material 2. [file 44313_2024_44_MOESM2_ESM.docx]

Supplementary Table S1.48 target genes in acute myeloid leukemia and myelodysplastic neoplasm panels

| Gene list | | | | |  |
| --- | --- | --- | --- | --- | --- |
| Acute myeloid leukemia | |  | Myelodysplastic neoplasm | | |
| *ABL1* | *KIT* |  | *ABL1* | *KRAS* | |
| *ANKRD26* | *KMT2A* |  | *ANKRD26* | *MECOM* | |
| *ASXL1* | *KRAS* |  | *ASXL1* | *MPL* | |
| *BCOR* | *MECOM* |  | *BCR* | *MYD88* | |
| *BRAF* | *MPL* |  | *BRAF* | *NF1* | |
| *CALR* | *MYD88* |  | *CALR* | *NPM1* | |
| *CBL* | *NF1* |  | *CBL* | *NRAS* | |
| *CDKN2A* | *NPM1* |  | *CEBPA* | *PDGFRA* | |
| *CEBPA* | *NRAS* |  | *CSF3R* | *PDGFRB* | |
| *CSF1R* | *PHF6* |  | *CXCR4* | *PTPN11* | |
| *CSF3R* | *PTPN11* |  | *DDX41* | *RAD21* | |
| *CXCR4* | *RAD21* |  | *DNMT3A* | *RUNX1* | |
| *DDX41* | *RARA* |  | *EPOR* | *SCF1R* | |
| *DNMT3A* | *RUNX1* |  | *ETV6* | *SETBP1* | |
| *ETV6* | *SETBP1* |  | *EZH2* | *SF3B1* | |
| *EZH2* | *SF3B1* |  | *FLT3* | *SMC1A* | |
| *FBXW7* | *SRSF2* |  | *GATA1* | *SMC3* | |
| *FLT3* | *STAG2* |  | *GATA2* | *SRSF2* | |
| *GATA1* | *TERT* |  | *IDH1* | *STAG2* | |
| *GATA2* | *TET2* |  | *IDH2* | *TERT* | |
| *IDH1* | *TP53* |  | *JAK2* | *TET2* | |
| *IDH2* | *U2AF1* |  | *JAK3* | *TP53* | |
| *JAK2* | *WT1* |  | *KDM6A* | *U2AF1* | |
| *JAK3* | *ZRSR2* |  | *KIT* | *ZRSR2* | |

Supplementary Table S2. Multivariate analysis of overall survival.

| Clinical variables | HR | (95% CI) | *P* |
| --- | --- | --- | --- |
| Age | 1.011 | (0.981-1.042) | 0.464 |
| WBC | 1.009 | (1.004-1.014) | 0.001 |
| Complex karyotypes^*^ | 0.949 | (0.363-2.483) | 0.916 |
| Allogenic HSCT^*^ | 0.322 | (0.107-0.969) | 0.045 |
| MR gene mutations^*^ | 1.391 | (0.659-2.935) | 0.389 |
| *TP53* mutations^*^ | 5.479 | (1.765-17.011) | 0.003 |

^*^The hazard ratio for the complex karyotype, allogenic HSCT, MR gene and *TP53* mutations indicates the relative risk of overall survival in cases with presence of these variables compared to absence.

Abbreviations: HR, hazard ratio; CI, confidence interval; WBC, white blood cell; HSCT, hematopoietic stem-cell transplantation; MR, myelodysplasia-related
